# Genetic and transcriptomic dissection of the fiber length trait from a cotton (*Gossypium hirsutum* L.) MAGIC population

**DOI:** 10.1186/s12864-019-5427-5

**Published:** 2019-02-06

**Authors:** Marina Naoumkina, Gregory N. Thyssen, David D. Fang, Johnie N. Jenkins, Jack C. McCarty, Christopher B. Florane

**Affiliations:** 1 Cotton Fiber Bioscience Research Unit, United States Department of Agriculture (USDA), Agricultural Research Service (ARS), Southern Regional Research Center (SRRC), 1100 Robert E. Lee Blvd, New Orleans, LA 70124 USA; 2 Cotton Chemistry and Utilization Research Unit, USDA-ARS-SRRC, 1100 Robert E. Lee Blvd, New Orleans, LA 70124 USA; 3 Genetics and Sustainable Agriculture Research Unit, USDA-ARS, 810 Highway 12 East, Mississippi State, MS 39762 USA

**Keywords:** Cotton, Fiber length, Genome-wide association study, Multi-parent advanced generation inter cross, Quantitative trait loci, RNAseq, Single nucleotide polymorphism

## Abstract

**Background:**

Improving cotton fiber length without reducing yield is one of the major goals of cotton breeding. However, genetic improvement of cotton fiber length by breeding has been a challenge due to the narrow genetic diversity of modern cotton cultivars and negative correlations between fiber quality and yield traits. A multi-parent advanced generation inter-cross (MAGIC) population developed through random mating provides an excellent genetic resource that allows quantitative trait loci (QTL) and causal genes to be identified.

**Results:**

An Upland cotton MAGIC population, consisting of 550 recombinant inbred lines (RILs) derived from eleven different cultivars, was used to identify fiber length QTLs and potential genes that contribute to longer fibers. A genome wide association study (GWAS) identified a cluster of single nucleotide polymorphisms (SNPs) on chromosome (Chr.) D11 that is significantly associated with fiber length. Further evaluation of the Chr. D11 genomic region among lines of the MAGIC population detected that 90% of RILs have a D11 haplotype similar to the reference TM-1 genome (D11-ref), whereas 10% of RILs inherited an alternative haplotype from one of the parents (D11-alt). The average length of fibers of D11-alt RILs was significantly shorter compared to D11-ref RILs, suggesting that alleles in the D11-alt haplotype contributed to the inferior fiber quality. RNAseq analysis of the longest and shortest fiber length RILs from D11-ref and D11-alt populations identified 949 significantly differentially expressed genes (DEGs). Gene set enrichment analysis revealed that different functional categories of genes were over-represented during fiber elongation between the four selected RILs. We found 12 genes possessing non-synonymous SNPs (nsSNPs) significantly associated with the fiber length, and three that were highly significant and were clustered at D11:24-Mb, including D11G1928, D11G1929 and D11G1931.

**Conclusion:**

The results of this study provide insights into molecular aspects of genetic variation in fiber length and suggests candidate genes for genetic manipulation for cotton improvement.

**Electronic supplementary material:**

The online version of this article (10.1186/s12864-019-5427-5) contains supplementary material, which is available to authorized users.

## Background

Cotton, *Gossypium* spp. is the major renewable source of fibers used worldwide in the textile industry. Cotton fibers are single-celled trichomes that emerge from ovule epidermal cells. About 25–30% of these epidermal cells differentiate into spinnable fibers [1, 2]. Cotton fiber development consists of four distinct but overlapping stages, including initiation, elongation, secondary cell wall biosynthesis and maturation [1, 2]. In cultivated cotton species, seed trichomes differentiate into two distinct types, long lint fibers that are easily detached from the seeds, and fuzz fibers that are short fibers strongly adhering to the seeds [3]. The lint fiber cells elongate for about 25 days post anthesis (DPA) depending on the cotton cultivar and growth conditions [4].

Lint fiber length is an important trait for the woven textile industry, since longer fibers can be more efficiently spun into yarn. The ultimate goal for cotton breeding is improving fiber quality characteristics without reducing fiber yield. However, this goal is challenging due to strong negative associations between lint length and yield in cotton [5–8]. The narrow genetic diversity of current cotton cultivars, due to thousands of years of domestication and selection, is also a limiting factor for breeding progress [9–13]. These problems can be partially overcome through generating populations from multiple parents, recombined over several generations. Such populations are termed Multi-parent Advanced Generation Inter Crosses (MAGIC) and offer great potential for improving breeding populations as well as for high-resolution trait mapping [14].

Fiber length is a complex trait that is controlled by multiple genes. The goal of this research is to identify genes regulating fiber length development. The first step in detecting gene-trait associations is often identifying quantitative trait loci (QTL). Linkage mapping using bi-parental populations has been a traditional approach for dissecting the genetic architecture of fiber quality traits [15–18]. These studies significantly improved our knowledge of cotton fiber genetics; however, most of these QTLs obtained from interspecific populations are often not stable across populations and not directly applicable to Upland cotton improvement [19]. Genome wide association studies (GWAS) have become a powerful tool that overcomes the limitations of bi-parental populations. A single GWAS can effectively associate genotypes with phenotypes in natural and man-made populations and can simultaneously detect candidate genes [20, 21]. In Upland cotton, GWAS have been successfully applied to identify QTLs and candidate genes for fiber quality traits [22, 23].

Previously, an Upland cotton MAGIC population was developed from eleven parental lines through five cycles of random mating followed by six cycles of self-pollination [24–26]. The effectiveness of random mating on population structure had been evaluated by SSR and SNP markers, and no obvious population structure was detected [24, 25]. The previous study [25] also conducted GWAS analysis of this MAGIC population using next-generation sequencing of genomic subsets targeted by restriction enzymes [27] for variant detection. That approach revealed more than 6000 SNP markers and subsequently lead to the detection of multiple QTLs for fiber quality traits across the *G. hirsutum* L. genome (including the Chr. D11 fiber length QTL) with a major QTL cluster on chromosome A07 [25]. In the current study, we used whole genome sequencing of 550 RILs for SNP identification. We detected a cluster of SNPs on Chr. D11 that was significantly associated with the fiber length. Further evaluation of the genomic sequences of RILs revealed that about 10% of the MAGIC population have the alternative Chr. D11 haplotype associated with shorter fiber length. The results of this study provide insights into the molecular aspects of genetic diversity of fiber length as well as potential candidate genes for fiber improvement.

## Results

### Genome wide association mapping revealed a significant fiber length QTL on Chr. D11

**Fig. 1:**
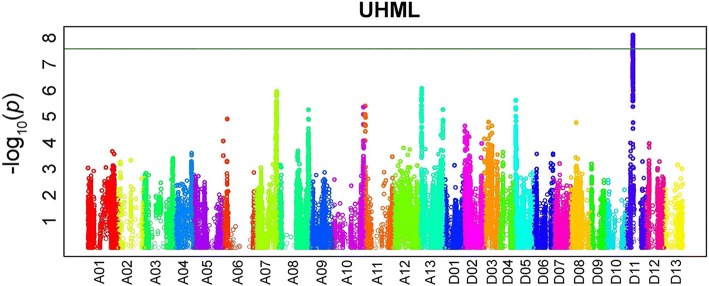
Manhattan plot for fiber length QTLs. UHML is Upper Half Mean Length. The negative log_10_ transformed *p* values were plotted against the marker positions of the physical map of each of the 26 chromosomes of *G. hirsutum* TM1. The significant threshold (−log10(*p) >* 7.6) is indicated by a green line

Previously, a fiber length QTL was identified in this MAGIC population on Chr. D11 by genotyping-by-sequencing of 6071 SNPs [25]. To further map this QTL region we used low coverage whole genome sequencing of 550 RILs to identify 473,517 SNPs [28]. Exploration of the genetic factors associated with fiber length in the MAGIC population was performed using GWAS with normalized fiber length values for 550 RILs grown in twelve environments. The best liner unbiased predictor (BLUP) was employed for normalization. A separate study, describing GWAS of fiber quality traits using this MAGIC population, has been reported elsewhere [28]. The Manhattan plot for fiber length trait was generated using GAPIT software and presented in Fig. 1. A cluster of 157 SNPs within the 250 kb region of Chr. D11 was significantly (Bonferroni correction) associated with fiber length in the MAGIC population (Fig. 1). GWAS also detected multiple peaks of SNPs across the *G. hirsutum* genome, which were less significantly associated with length trait than very conservative Bonferroni corrected threshold (Fig. 1).

### A sub-population of MAGIC RILs carrying the alternative Chr. D11 haplotype has significantly shorter fiber length

**Fig. 2:**
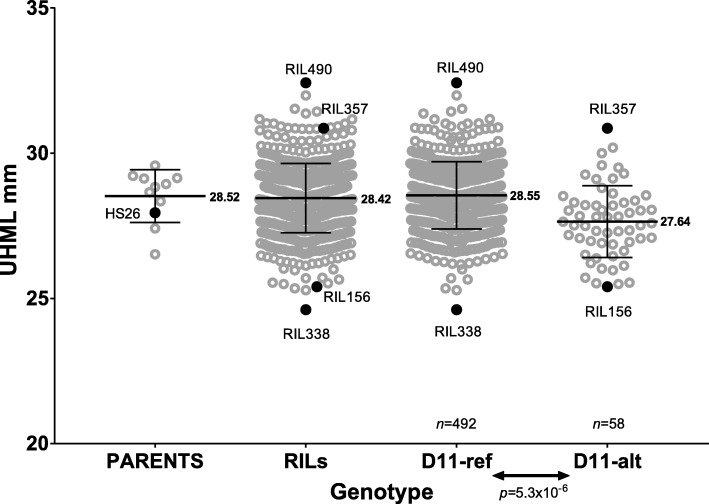
Phenotypic differences in fiber length between MAGIC subpopulations of RILs possessing D11-ref and D11-alt haplotypes. UHML is Upper Half Mean Length. Black lines indicate mean values of fiber length with numerical value at right. Error bars are standard deviation within tested populations. Significant difference in fiber length between D11-ref and D11-alt MAGIC populations was detected by a parametric (two-tailed) t-test. Grey circles represent normalized fiber length for each individual RIL measured at twelve different environments [24–26]; black dots represent the parent (HS26) that contributed the D11-alt haplotype and the longest and shortest RILs from both populations which were selected for RNAseq analysis

We evaluated differences in the Chr. D11 genomic sequence region (250 kb) among 550 RILs and detected that about 90% (*n* = 492) of the RILs have a D11 haplotype similar to the reference TM-1 genome (D11-ref), whereas 10% (*n* = 58) of RILs inherited an alternative haplotype (D11-alt) from parental cultivar HS26. Two other parent cultivars, Acala Ultima and FM966, also carried the D11-alt segment in a heterogenous state. Statistical evaluation of differences in fiber length between MAGIC sub-populations carrying D11-ref and D11-alt haplotypes revealed that RILs with D11-alt have significantly (*p* = 5.3 × 10^− 6^) shorter fiber length (Fig. 2). To get insights into the molecular mechanisms of fiber length we selected the longest and shortest fiber RILs from D11-ref (RIL490 and RIL338) and D11-alt (RIL357 and RIL156) sub-populations for transcriptional analysis of developing fibers (Fig. 2).

### Fiber quality analysis

Plants from the four selected RILs along with the parents were grown during the summer of 2017 in USDA-ARS field plot in New Orleans, LA, USA. Fiber quality measurements were made using an Advanced Fiber Information System (AFIS) instrument. The measurements obtained from mature fibers of the four RILs and their parents revealed that RIL490 produced the longest fiber (Table 1). The short fiber content was lowest for ST825 (2.3 + 0.52) and highest for M240 (5.8 + 3.64). RIL490, FM966 and RIL357 produced the finest fiber (considering the range of standard deviations, Table 1). All lines produced mature fiber with a maturity ratio about 0.9. Micronaire (MIC) represents a combination of fiber maturity ratio and fineness. The premium MIC readings (3.97 + 0.06) as determined by the Fibronaire instrument were observed only for RIL490. The results of fiber length measurements of the tested lines were consistent with data previously collected by a High Volume Instrument (HVI) from plants grown in twelve different environments. The RIL490 produced the longest fiber regardless of the growth environment, suggesting a major influence on this trait by genetic factors.

**Table 1:** AFIS and Fibronaire measurements of fiber traits of selected MAGIC RILs and parents grown in New Orleans field, 2017

Genotype	Length, mm	Rating	Short fiber content, %	Fineness, (millitex)	Rating	Maturity ratio	Micronaire	Rating
156_RIL	24.47 ± 0.78	Short	2.73 ± 0.31	186 ± 13	Average	0.93 ± 0.03	5.43 ± 0.26	Discount
338_RIL	23.88 ± 0.44	Short	3.23 ± 0.6	185 ± 8	Average	0.92 ± 0.01	5.56 ± 0.15	Discount
357_RIL	29.46 ± 0.67	Long	2.77 ± 0.31	167 ± 3	Fine	0.94 ± 0.02	4.81 ± 0.08	Base
**490_RIL**	**32.17 ± 0.89**	**Extra long**	**3.3 ± 0.53**	**162 ± 6**	**Fine**	**0.9 ± 0.03**	**3.97 ± 0.06**	**Premium**
551_ACALA	28.96 ± 1.16	Long	3.1 ± 0.2	158 ± 24	Average	0.92 ± 0.06	4.29 ± 0.05	Base
552_PYRAMID	25.74 ± 0.53	Short	3.13 ± 0.76	191 ± 7	Average	0.92 ± 0.01	5.26 ± 0.06	Discount
553_COKER	26.08 ± 0.53	Medium	3.4 ± 0.46	184 ± 5	Average	0.94 ± 0.01	4.95 ± 0.2	Discount
554_ST825	27.35 ± 0.39	Medium	2.3 ± 0.52	193 ± 3	Average	0.95 ± 0.02	5.26 ± 0.11	Discount
555_FM966	28.53 ± 0.78	Long	3.43 ± 0.32	157 ± 13	Fine	0.93 ± 0.04	4.67 ± 0.06	Base
556_M240	22.44 ± 1.03	Short	5.8 ± 3.64	179 ± 23	Average	0.88 ± 0.06	5.26 ± 0.06	Discount
557_HS26	25.82 ± 1.2	Short	4.23 ± 1.65	172 ± 12	Average	0.88 ± 0.03	4.95 ± 0.23	Base
558_DP90	26.75 ± 0.53	Medium	2.63 ± 0.6	168 ± 13	Average	0.9 ± 0.04	4.93 ± 0.09	Base
559_SG747	27.01 ± 0.82	Medium	2.7 ± 0.82	178 ± 5	Average	0.88 ± 0.02	5.24 ± 0.17	Discount
560_PSC355	27.18 ± 0.25	Medium	2.97 ± 0.93	185 ± 9	Average	0.88 ± 0.04	5.18 ± 0.05	Discount
561_STV474	27.86 ± 0.15	Medium	2.5 ± 0.1	179 ± 9	Average	0.9 ± 0.02	4.83 ± 0.09	Base

Ratings of fiber traits were provided according to standards available at Cotton Incorporated web site (http://www.cottoninc.com/fiber/quality/US-Fiber-Chart/Ratings-Of-Fiber-Properties/). Standard deviations provided for 3 technical replicates. The best fiber properties for RIL490 are highlighted by bold font

### Transcriptional analysis of developing fibers of longest and shortest RILs carrying different Chr. D11 haplotypes

**Fig. 3:**
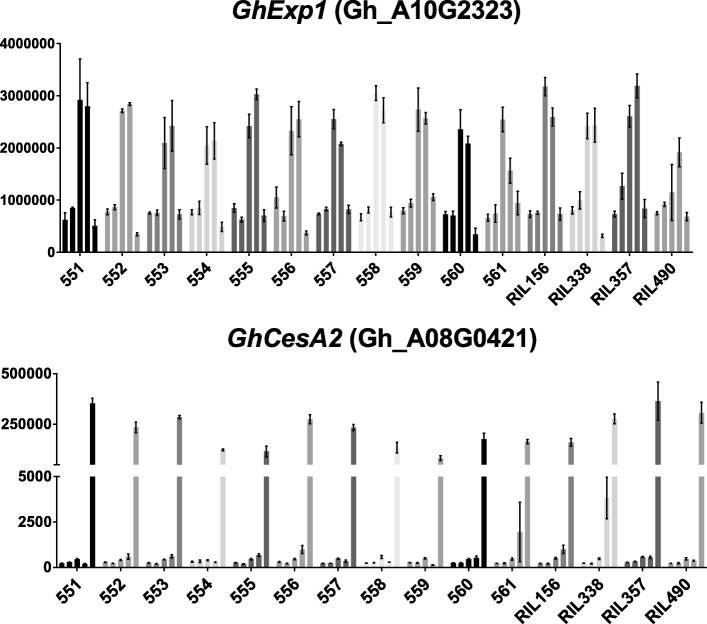
Preliminary RT-qPCR analysis of developing fibers of selected RILs and parents. Vertical axis represents relative gene expression normalized by 18S rRNA. Different shade bars represent fiber developmental time points in increasing order of 3, 5, 8, 12 and 16 DPA. Parental lines are listed in increasing number 551 to 561, their names are provided in Table 1. NBI *G. hirsutum* TM-1 genome gene numbers are shown in parentheses for each gene. Error bars indicate the standard deviation from three biological replicates

Genome-wide gene expression was evaluated by RNAseq in elongating fiber cells of selected RILs and their parents. Preliminary RT-qPCR was conducted to determine peak of fiber elongation and transition to secondary cell wall (SCW) biosynthesis in the selected lines. The elongation stage related gene *GhExp1* [29] and secondary cell wall biosynthesis stage related gene *GhCesA2* [30] were used as control genes. Figure 3 represents the expression patterns of *GhExp1* and *GhCesA2* genes during the fiber developmental time course of 3, 5, 8, 12 and 16 DPA in 4 RILs and parents. The peak of *GhExp1* expression was 8–12 DPA for all tested lines. Interestingly, *GhExp1* reached maximal activity later at 12 DPA in the two longest fiber lines RIL357 (D11-alt) and RIL490 (D11-ref) comparing to 8 DPA in their short fiber analogues. A prolonged elongation period in these RILs may contribute to longer fiber [31]. The expression level of *GhExp1* was about two fold less at 8 DPA in the longest fiber RIL490 compared to the other lines (Fig. 3). The transition to SCW biosynthesis occurred at 16 DPA in all tested lines. The time point 8 DPA was selected for RNAseq analysis.

**Fig. 4:**
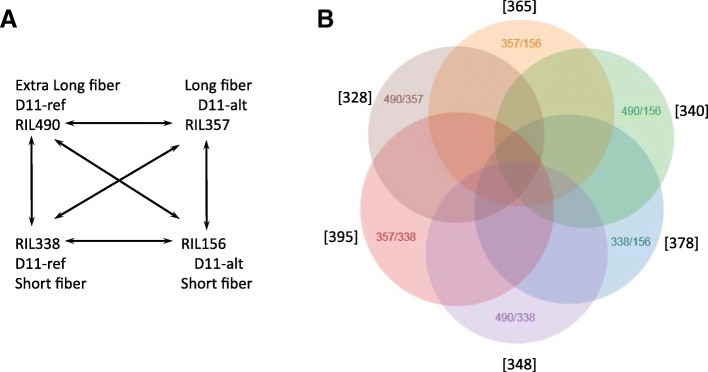
Differential gene expression analysis. **a** Design of comparisons between selected RILs. **b** Venn diagram of significantly regulated genes between RILs

Forty-five RNA samples from four RILs and eleven parents (three biological replicates each) were Illumina sequenced with over 100 million raw reads per sample. Approximately 55 to 68% of clean reads were mapped to predicted coding sequences of the NBI *G. hirsutum* TM-1 genome (Additional file 1) [32]. Reads were mapped only to coding sequences of predicted proteins. Therefore, the percentage of mapped reads were lower than mapping to the whole genome (which includes UTRs, introns, long non-coding RNAs, transposons, etc.). An ANOVA model was utilized to identify Differentially Expressed Genes (DEGs) between four RILs in six possible comparisons (Fig. 4a). We found that 949 genes in total were significantly (FDR < 0.05) differentially expressed between four RILs in six comparisons. Additional file 2 provides ANOVA analysis results. Quantities of DEGs between comparisons were relatively similar, from 328 to 395 genes (Fig. 4b).

**Fig. 5:**
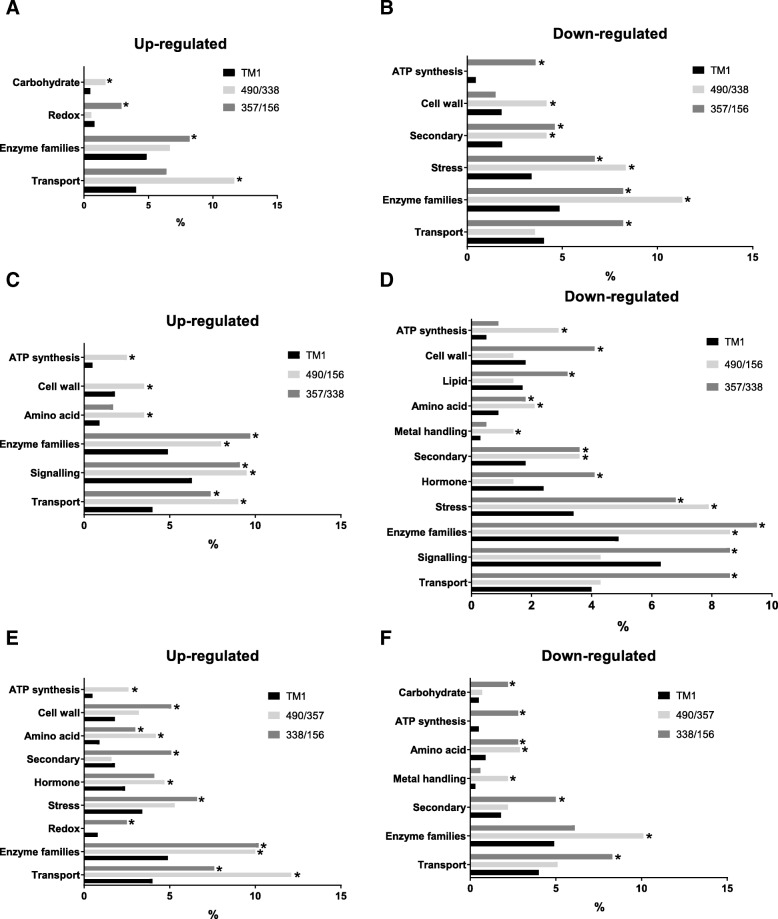
Gene set enrichment analysis of DEGs in longest and shortest fiber RILs carrying different D11 haplotypes. MapMan ontology was used for functional characterization of DEGs; only significantly (*p* < 0.05; asterisks) over-represented categories are shown. Up-regulated (**a**, **c**, **e**) and down-regulated (**b**, **d**, **f**) gene families in comparisons between RILs 490/338, 357/156, 490/156, 357/338, 338/156 and 490/357. Relative gene frequencies in functional categories are presented in percent from amount of up-regulated or down-regulated genes; background represents the NBI *G. hirsutum* TM-1 reference genome

To evaluate the functional distribution of DEGs, MapMan ontology was used for gene Set Enrichment Analysis (SEA) [33]. SEA revealed that different functional categories of genes were over-represented in four RILs at 8 DPA fiber elongation (Fig. 5). For example, comparisons between the longest and shortest fiber RILs carrying the same haplotype RILs 490/338 (D11-ref) and RILs 357/156 (D11-alt) revealed very few functional categories of genes were significantly (*p* < 0.05) over-represented among the up-regulated genes. Particularly, carbohydrate metabolism and transport were over-represented in RILs 490/338, whereas redox and enzyme families were over-represented in RILs 357/156 (Fig. 5a). Larger quantity of functional categories of genes, including those involved in ATP synthesis, cell wall, secondary metabolism, stress, enzyme families and transport, were over-represented among down regulated DEGs in long versus short fiber lines (Fig. 5b). Comparisons between longest and shortest fiber RILs carrying different D11 haplotypes revealed that more gene families were over-represented among up-regulated and down-regulated genes relative to comparisons between lines with the same haplotype (Fig. 5). In addition, comparisons between longest RILs 490/357 and shortest RILs 338/156 carrying different D11 haplotypes confirmed the same observation.

### Non-synonymous SNPs associated with fiber length

Nearly eight thousand non-synonymous SNPs (nsSNPs) were identified in the coding regions of annotated proteins [32]. Among them, twelve genes were identified in significantly (Chr. D11) or non-significantly (Chr. A07, A08, A11, D02, D05 and D12) Bonferroni corrected QTLs. Table 2 provides the name of genes, the SNPs and their positions, average fiber length of MAGIC sub-populations carrying specific SNP and significance of the association with fiber length.

**Table 2:** Non-synonymous SNPs associated with fiber length

Gene	Position	Ref	Ref aa	REF, n	Ref, mm	Alt	Alt aa	ALT, n	Alt, mm	*P*-value
Gh_A07G1744	71,156,748	G	V	460	28.4	A	M	99	28.9	1.4E-05
Gh_A07G1769	72,203,768	T	K	475	28.4	C	E	84	29.0	3.4E-06
Gh_A07G1793	72,731,159	A	E	427	28.3	T	D	132	28.8	7.1E-05
Gh_A08G1774	97,828,015	A	K	451	28.6	T	M	108	28.0	4.0E-06
Gh_A08G1775	97,852,975	C	R	446	28.6	G	G	113	28.0	4.6E-07
Gh_A11G0352	3,230,793	T	N	437	28.6	A	Y	122	28.0	2.6E-07
Gh_D02G0366	4,864,217	G	A	487	28.4	C	G	72	28.9	2.9E-04
Gh_D05G0610	4,932,816	T	R	362	28.7	C	K	198	28.1	6.8E-08
Gh_D11G1928	24,009,361	G	R	495	28.5	A	C	63	27.8	5.3E-06
Gh_D11G1929	24,030,087	G	E	505	28.5	T	D	54	27.8	1.2E-05
Gh_D11G1931	24,065,685	A	K	499	28.5	G	R	60	27.8	1.0E-06
Gh_D12G0592	11,043,241	T	V	514	28.5	C	I	45	27.7	1.3E-05

Column names of the table represent reference (Ref) to NBI *G. hirsutum* TM-1 genome and alternative (Alt) SNP and amino acid (aa); number (n) of RILs with Ref or Alt SNPs; average length in mm RILs carrying Ref or Alt SNPs. *P*-values were determined by two-tailed t-test

**Fig. 6:**
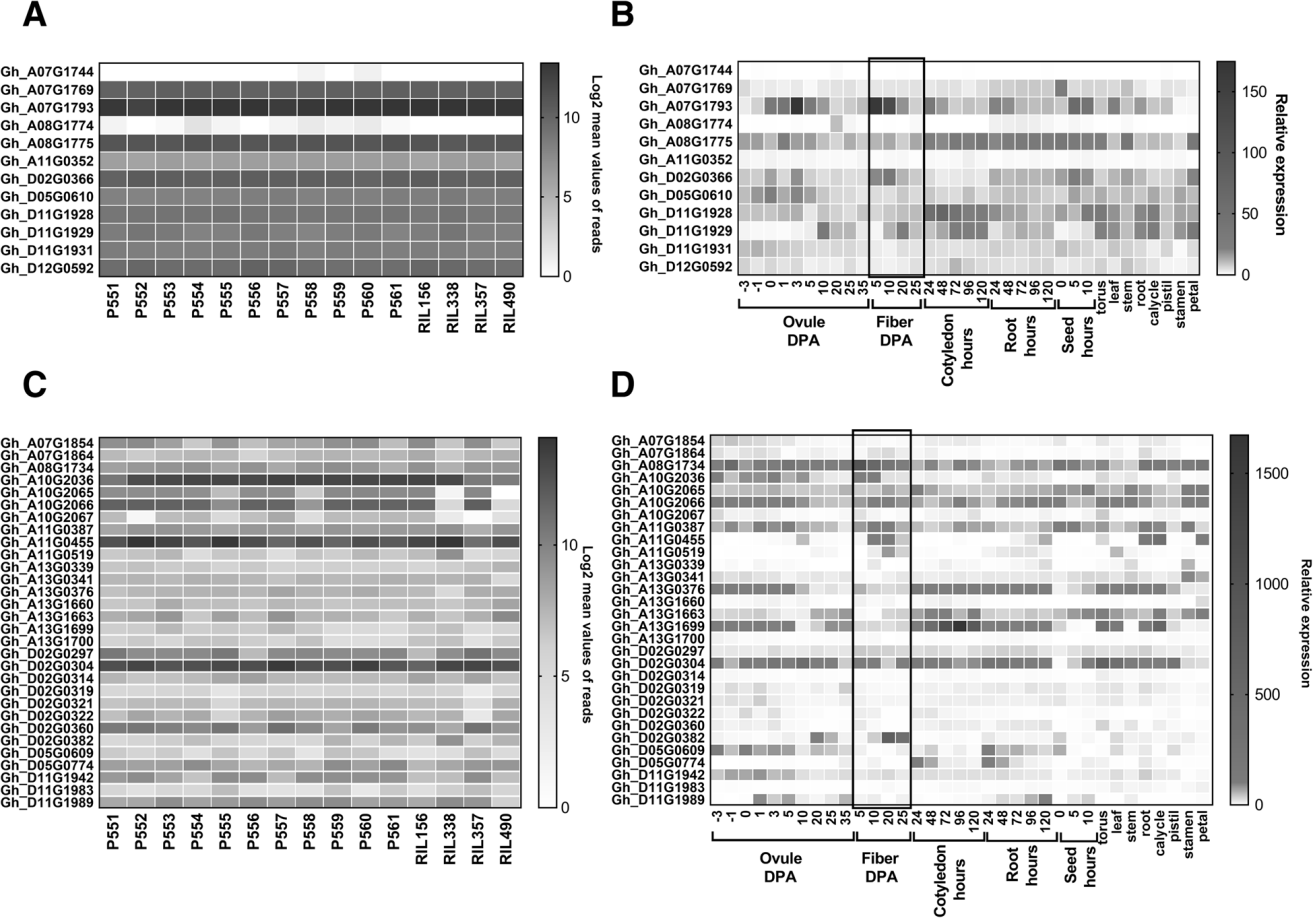
Heat maps of gene expression. Expression patterns of genes carrying non-synonymous SNPs associated with fiber length in MAGIC parents and four RILs at the 8 DPA fiber development stage (**a**) and in different cotton tissues (**b**). Expression patterns of DEGs near fiber length QTLs length in MAGIC parents and four RILs at 8 DPA (**c**) and in different cotton tissues (**d**). Expression data for different cotton tissues were obtained from ccNET database (http://structuralbiology.cau.edu.cn/gossypium/). Fiber developmental tissues were marked with a rectangle

We examined expression levels of these twelve genes in RNAseq data of the four RILs and eleven parents. The expression levels of these genes were not significantly different between the lines at 8 DPA and expression was not detected for two genes (Gh_A07G1744 and Gh_A081774) in developing fibers (Fig. 6a). The expression of these genes were also evaluated in different tissues from the publically available ccNET database at http://structuralbiology.cau.edu.cn/gossypium/ [34]. Gh_A081774 showed weak expression in ovules at 20 DPA. Five genes including Gh_A07G1793 (RAB GTPase), Gh_A08G1775 (Zinc finger C3HC4-type transcription factor), Gh_D02G0366 (unknown), Gh_D11G1928 (methionine aminopeptidase) and Gh_D11G1929 (KIP-related protein 6) are expressed in developing fibers (Fig. 6b).

### DEGs near fiber length QTLs

Thirty of the 949 DEGs were identified within one million base pairs of the fiber length QTLs. Additional file 3 provides coordinates of SNPs which were considered in selection of nearest DEGs, whereas Additional file 2 provides coordinates of genes and their expression. Expression patterns of these 30 genes were examined in RNAseq data of the four RILs and parents and in different cotton tissues obtained from the ccNET database (Fig. 6c and d). Two of the most highly expressed genes Gh_A10G2036 and Gh_A11G0455 showed a significant reduction of expression in longer fiber RILs 357 (D11-alt) and 490 (D11-ref) at 8 DPA fiber development. Gh_A10G2036 is preferentially expressed in early developing ovules and fiber cells, whereas Gh_A11G0455 is preferentially expressed in fiber cells, ovules at 10 DPA, and root, calycle and petal tissues (Fig. 6d). Gh_A10G2036 is annotated as ROP guanine nucleotide exchange factor five, while Gh_A11G0455 is xyloglucan endotransglycosylases/hydrolase-1 (*GhXTH1*).

Three DEGs were identified near the D11 QTL. Two of them, Gh_D11G1942 and Gh_D11G1983, are annotated as protein-kinase superfamily proteins, whereas Gh_D11G1989 is annotated as auxin-responsive GH3 family protein. Two DEGs, Gh_D11G1942 and Gh_D11G1989, were expressed in ovules and fiber cells (Fig. 6d). Only Gh_D11G1989 showed significant expression differences in the longest fiber RIL490 compared to the other lines (Fig. 6c), which suggests that this gene may regulate fiber length in RIL490. However, we identified 14 MAGIC RILs with recombinations between D11:24-Mb and Gh_D11G1989 (Additional file 4), indicating that this gene is unlikely to itself harbor the causative D11 QTL polymorphism.

### RNA expression analysis of candidate genes from D11 QTL

**Fig. 7:**
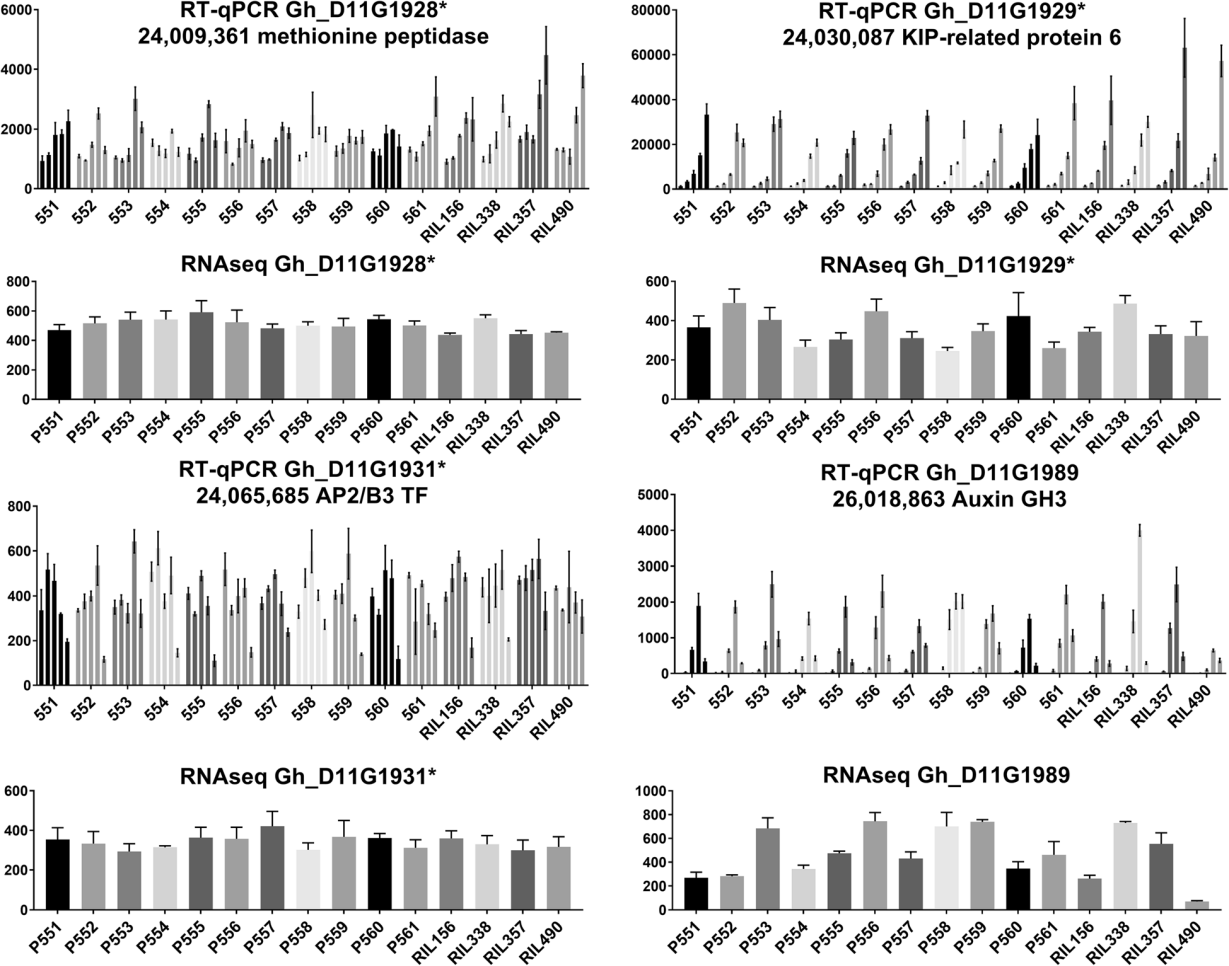
RNA expression analysis of genes associated with the D11 fiber length QTL. Vertical axis represents relative gene expression normalized by 18S rRNA for RT-qPCR analysis and number of reads detected by RNAseq. Different shade bars represent fiber developmental time points in increasing order 3, 5, 8, 12 and 16 DPA; only 8 DPA was used for RNAseq. Numbers next to the gene name indicates position on chromosome. Asterisks indicate nsSNP between RILs in the gene

Furthermore, expression patterns of four selected genes (three with nsSNPs and one DEG) from the D11 QTL were examined by RT-qPCR analysis of the parents and four RILs in developing fibers at 3, 5, 8, 12 and 16 DPA (Fig. 7). The results of RT-qPCR were consistent with the results of RNAseq analysis (Fig. 7). Methionine aminopeptidase (Gh_D11G1928) and KIP-related protein 6 (Gh_D11G1929) showed the highest expression level during the transition to SCW biosynthesis at 16 DPA. Similar expression patterns during fiber development were observed for Gh_D11G1929 in the ccNET database (Fig. 6b) [34]. At 16 DPA, both of these genes had significantly higher expression in longer fiber RILs 357 and 490 compared to shorter fiber lines 156 and 338 (Fig. 7). Gh_D11G1931, annotated as AP2/B3 transcription factor, did not show significantly different expression between RILs either by RNAseq or RT-qPCR analysis. Gh_D11G1989, a DEG near the D11 QTL, showed the highest expression during the peak of fiber elongation at 8–12 DPA (Fig. 7). Also, according to ccNET data, this gene was highly expressed in early developing ovules and root tissue, which would suggest involvement of this gene in fiber development and probably in root cell elongation (Fig. 6d). RT-qPCR data for this gene was consistent with RNAseq analysis and confirmed that expression of Gh_D11G1989 was significantly lower in RIL490 compared to other lines.

## Discussion

Using GWAS of a MAGIC population we found a highly significant QTL on Chr. D11 as well as a number of less significant QTLs associated with fiber length. The same fiber length QTL on Chr. D11 was previously identified in our lab using fewer markers [25] and by two independent research studies using GWAS analysis of 719 and 419 diverse accessions of Upland cotton [22, 23]. These studies, which used GWAS of diverse accessions of Upland cotton, identified the same fiber length candidate genes on Chr. D11 as we have, including Gh_D11G1928, Gh_D11G1929 and Gh_D11G1931 [22, 23]. Information from four independent studies increases the likelihood of the QTL linkage relationship. Some of the prominent GWAS peaks we observed, that were below the conservative Bonferroni correction, should also be considered for cotton QTL meta-analysis [35].

Sequence evaluation of the Chr. D11 QTL haplotype in the 550 RILs revealed that 10% of MAGIC population inherited the D11-alt segment from the HS26 parental line (also cultivars Acala Ultima and FM966 carried D11-alt segment in a heterogeneous state). Possessing the D11-alt chromosomal segment was significantly associated with shorter fiber length among RILs in the MAGIC population (Fig. 2). Interestingly, the HS26 parental line is not the line which produces the shortest fibers among the parents used for the creation of the MAGIC population. Depending on the year, HS26 produces medium or short cotton fibers, while the two heterogeneous parental lines usually produce long cotton fiber. The less significant fiber length QTLs may control fiber length in HS26. In addition, there are long cotton fiber producing lines observed among RILs carrying the D11-alt haplotype. Such variations in fiber length among sub-populations can be explained by the genetic complexity of the trait.

One of the goals of QTL analysis is to determine whether the phenotype is controlled by a few loci with strong effect or by multiple loci with weak effects. The QTLs with strong effects on phenotype will exhibit Mendelian segregation. In our study, GWAS analysis identified a significant fiber length QTL on Chr. D11. Whether this QTL has strong effect on fiber length will be determined in future studies, by mapping an F_2_ population derived from crosses of RILs possessing D11-ref and D11-alt loci.

To get insights into the genetic control of fiber length we conducted comparative RNAseq analysis of the longest and shortest RILs from D11-ref and D11-alt sub-populations. Noticeably, larger quantities of enriched functional categories of genes were detected between comparisons of RILs possessing different D11 haplotypes than between comparisons of RILs possessing the same haplotype. This observation suggests that genetic differences in D11 haplotypes are the reason for different distributions of significantly enriched gene families in the tested RILs.

We identified 12 genes having nsSNPs in protein coding regions in QTLs associated with fiber length trait. None of them showed significantly different expression between the select RILs or among the parental lines at 8 DPA of fiber development. It should be noted that non-synonymous substitutions in protein coding regions may not necessarily alter the transcript abundance of the gene. However, amino acid point mutations may change protein structure and function.

Furthermore, we found 30 DEGs in close proximity to fiber length QTLs. Two highly induced genes, Gh_A10G2036 (QTL A10) and Gh_A11G0455 (QTL A11), showed specific reduction of expression in the two longest RILs, 357 and 490 (Fig. 6). The ROP guanine nucleotide exchange factor five (Gh_A10G2036) belongs to kinase partner protein-like gene family, which controls many eukaryotic cellular processes, including Rho GTPase-dependent polar growth [36]. The role of this gene in cotton fiber development is unknown. Gh_A11G0455 is xyloglucan endotransglycosylases/hydrolase-1 (*GhXTH1*), which has been functionally characterized in cotton. Plants that over-expressed *GhXTH1* produced 15–20% longer fiber [37].

Four potential candidates of fiber-length-related genes from the Chr. D11 QTL will be discussed here in more detail. Three genes from the D11 locus, including Gh_D11G1928, Gh_D11G1929 and Gh_D11G1931, contain nsSNPs. Another gene Gh_D11G1989 (in close proximity to D11 locus) showed significant transcriptional differences in the longest fiber RIL490.

Two genes with nsSNPs showed increased expression in the longest fiber RILs at 16 DPA. Gh_D11G1928 is annotated as a methionine aminopeptidase. The function of this gene in cotton is unknown. The Arabidopsis homolog of this gene (At4G37040) is involved in N-terminal protein amino acid modification, proteolysis and response to abscisic acid [38]. Abscisic acid has a negative effect on cotton fiber elongation; it is well documented that exogenous application of abscisic acid inhibits growth in an in vitro cotton ovule-culture system [39, 40]. Gh_D11G1929 is annotated as KIP-related protein 6. In plants, KIP-related proteins (KRP) are negative regulators of cell division, by inhibiting cyclin-dependent kinases [41]. Elevated levels of KRP6 in Arabidopsis negatively affected plant development and fertility [42]. However, some members of the KRP family in Arabidopsis showed multiple functions. For example, KRP5 controls endoreduplication that allows expression of genes required for cell elongation [43]. Overexpression of cotton Gh_D11G1929 in Arabidopsis resulted in about 2-fold longer leaf trichomes; though, the number of leaf trichomes decreased about 2-fold in transgenic lines [22]. The function of Gh_D11G1929 in cotton fiber development is unknown. Elevated expression of these two genes in the longer fiber RILs suggests their involvement in the fiber development process.

The function of Gh_D11G1931, annotated as AP2/B3-like transcriptional factor, is unknown in cotton. Although no significant difference in expression between tested RILs (Fig. 7), this gene should not be ignored as potential candidate due to a nsSNP in the C-terminal domain of this transcription factor (Table 2).

In close proximity to the D11 QTL we found a significantly differentially expressed gene Gh_D11G1989, annotated as an auxin-responsive GH3 family protein. Gh_D11G1989 exhibited a typical fiber-elongation pattern in all tested lines with a maximal peak of expression at 8–12 DPA (Fig. 7). This gene showed a unique reduction of expression in the longest fiber line RIL490 (D11-ref allele) that was detected consistently by RNAseq and RT-qPCR techniques. The function of Gh_D11G1989 is unknown in cotton. Multiple GH3 proteins, mainly from Arabidopsis and rice, have been functionally characterized and showed similar biochemical activity of Indole-3-acetic acid (IAA)-amido synthetases that conjugate amino acids to IAA [44–46]. Auxins, primarily IAA, are involved in plant growth and developmental processes, including cell division, elongation and differentiation. In addition to the free acid, IAA occurs in a variety of conjugated forms with various glycosyl esters, amino acids and peptides. Only free IAA is biologically active, but its conjugates help to maintain IAA homeostasis, by preserving IAA until it is needed. High sensitivity of GH3 genes to exogenous application of auxins may explain how plants cope with excess of auxin. Transcriptional activation of GH3 gene would lead to accumulation of more IAA-amido synthetase, which then converts excess auxin to amino acid conjugates that are either inactive or degraded. It has been shown experimentally that overexpression of GH3–8 in rice retarded plant growth and development [46]. We speculate that low transcriptional activity of Gh_D11G1989 in RIL490 during fiber development may leave a large amount of free active IAA which contributes to longer fiber. We suggest that Gh_D11G1989 is critical for the longer fiber associated with the D11 QTL; though, the causative factor that controls transcript abundance of this gene is still unknown. We speculate that the expression level of Gh_D11G1989 might be directed by an unknown regulatory element within the D11:24 QTL region that contributes to longer fiber cells in RIL490. Although this gene is outside of the GWAS D11 QTL peak by 1-Mb (Additional file 4), causative mutations have been reported at more than 350-kb from the affected genes that in turn, influence the phenotype [47].

In future studies, we plan to perform fine mapping of the F_2_ progeny of RIL490 x RIL156 crosses, which may help identify the causative gene of D11 QTL. It should be mentioned that the reference genome used in this study [32] has many scaffolds that are not assigned to chromosomes. The causative D11 QTL gene may be among these scaffolds. The improved reference genome will be used for fine mapping as soon as it released to the public.

## Conclusions

We have identified a highly significant QTL on chromosome D11 as well as a number of less significant QTLs associated with fiber length by using whole genome sequencing and GWAS of a MAGIC population. Ten percent of the MAGIC population inherited, from one of the parents, the D11-alt haplotype associated with shorter fiber length. RNAseq analysis of the longest and shortest RILs from D11-ref and D11-alt sub-populations helped to detect potential candidate genes. We suggest that low transcriptional activity of the auxin-responsive GH3 gene (Gh_D11G1989) in the longest fiber line (RIL490) may result in a greater amount of active IAA, which contributes to longer fiber. The results of this study provide insights into molecular control of fiber length and candidate genes for genetic manipulation for cotton improvement.

## Methods

### Plant materials and field experiments

The development of this MAGIC population was described before in details [24–26]. Briefly, a half-diallel crossing scheme between eleven parents was followed to produce 55 F_1_ in 2002. The 55 F_1_ were considered as 55 half-sib families and designated as Cycle 0 (C_0_). Five cycles (C_1_ to C_5_) of random mating were made by bulking an equal amount of pollen from each of the 55 families. After that, self-pollination was followed for six generations using single seed descent to produce RILs. The MAGIC population of 550 RILs was composed from ten RILs from each of the 55 families [25].

The seeds of 550 RILs along with their eleven parents were planted as two replicates in a randomized complete block design in fields of Florence, SC in 2014, 2015 and 2016, Starkville, MS in 2009, 2010, 2011, 2014, 2015, and 2016, and in Stoneville, MS in 2013, 2014 and 2015. Each plot was 12 m long with about 120 plants. Standard field practices were applied over the plant growing seasons across years and locations. Twenty-five naturally opened bolls were harvested manually from the central part of a plant for fiber quality testing. Phenotypic data was normalized using a best linear unbiased predictor (BLUP) implemented in R software [28].

In this study, we used eleven parents and four RILs for ovule collection. Selection of the four RILs for this study is explained in the text. About 50 plants from each of the parents and four RILs were grown during the summer of 2017 in a field in New Orleans, LA. Plants from each line were labeled into three pools representing three biological replicates. Cotton bolls were harvested at the following time-points during the initiation and early, peak and late elongation stages of cotton fiber cell development: 3, 5, 8, 12, and 16 days post anthesis (DPA). The number of bolls per bulked sample varied according to developmental time-point, with a greater number of bolls required for the earliest time-point. For example, ovules from approximately 20–30 bolls were bulked for each 3 DPA sample, and ovules with attached fibers from approximately 8–10 bolls were bulked for each 16 DPA sample. Harvested bolls were placed immediately on ice and transported to the laboratory where they were dissected on ice, frozen in liquid nitrogen and stored at − 80 °C. For fiber testing, 25 naturally opened bolls were manually harvested from the central part of the plant.

### Fiber quality measurements

For mature fiber quality measurements the cotton boll samples were ginned using a laboratory saw gin. Fiber quality attributes of MAGIC population samples grown in Florence, Starkville and Stoneville during different years were measured using a High Volume Instrument (HVI, USTER technologies Inc., Charlotte, NC). HVI data was collected for different traits [25]; in this study we used upper half mean length (UHML) fiber measurements for genome wide association analysis.

The fiber testing data of New Orleans grown plants were determined by the Cotton Fiber Testing Lab, USDA-ARS-SRRC, New Orleans, LA. Micronaire (MIC) values were measured by the Fibronaire instrument (Motion Control Inc., Dallas, TX). A higher MIC value indicates a more mature, coarser fiber. Fiber mean length, short fiber content (SFC), fineness, and maturity ratio were measured by the Advanced Fiber Information System (AFIS) (USTER Technologies Inc.). Fiber mean length is the average length of a fixed weight of fibers expressed in mm. SFC is the percent of fibers less than 12.7 mm in a fixed weight of fibers. Fiber fineness is given as millitex, which is a measure of linear density derived by the weight of fibers in micrograms per length of fibers in meters. Maturity ratio indicates the fiber maturity in terms of the degree of thickening of the SCW relative to the diameter or fineness of the fiber. A higher maturity ratio indicates a more mature fiber.

### Nucleic acids isolation and RT-qPCR

Young leaves were collected from parental cultivars and 550 RILs. DNA was isolated as previously described [48]. The concentration of DNA samples was measured using a NanoDrop 2000 spectrophotometer (Thermo Fisher Scientific, Waltham, MA), whereas quality was estimated on 1.5% agarose gel.

Cotton fibers were isolated from developing ovules using a glass bead shearing technique to separate fibers from the ovules [49]. Developing fibers from 12 to 16 DPA ovules were manually separated by forceps. Total RNA was isolated from detached fibers using the Sigma Spectrum Plant Total RNA Kit (Sigma-Aldrich, St. Louis, MO) with the optional on column DNase1 digestion according to the manufacturer’s protocol. The concentration of each RNA sample was determined using a NanoDrop 2000. The RNA quality for each sample was determined by RNA integrity number (RIN) using an Agilent Bioanalyzer 2100 and the RNA 6000 Nano Kit Chip (Agilent Technologies Inc., Santa Clara, CA) with 250 ng of total RNA per sample. A detailed description of reverse transcription and qPCR for quantification of mRNA transcripts was previously reported [50, 51]. 18S rRNA was used as the endogenous reference gene for relative quantitation of the gene expression data. The relative expression levels were calculated using the 2^−ΔΔCt^ method [52]. Sequences of primers are listed in Additional file 5.

### Genome sequence and genome wide association study

Genome sequence analysis of MAGIC population and genome wide association study (GWAS) methodology for different fiber traits is described elsewhere in detail [28, 53]. Briefly, the whole genomes of eleven MAGIC parents were sequenced at 20× coverage and all 550 RILs were sequenced at 3× coverage with Illumina short read paired-end sequencing. Genome sequencing was conducted by Novogene Corporation (Chula Vista, CA, USA) using Illumina HiSeq 2500 equipment. Sequence reads were aligned to the NBI *G. hirsutum* cv TM-1 reference genome [32] with GSNAP software [54]. SNPs were identified with samtools and bcftools software [55]. About half million polymorphic SNPs that met filtering criteria were used for GWAS. GAPIT software with default parameters [56] was used to determine the association between SNPs and fiber length using a mixed linear model (MLM) [57].

The significance of the associations between SNPs and traits was based on the threshold of the Bonferroni correction for multiple tests.

### RNAseq and data analyses

RNA samples from three biological replicates of developing cotton fibers at 8 DPA from MAGIC parental lines and four RILs were subjected to paired-end Illumina (Platform PE150) mRNA sequencing (RNAseq). The time point 8 DPA was selected because it represents the peak of fiber cell elongation. Library preparation and sequencing were performed by Novogene Corporation (Chula Vista, CA, USA). Mapping the reads to the *G. hirsutum* TM-1 coding DNA sequences (CDS) [32] was performed using GSNAP software [54]. Default parameters were used, but with the flags “-n 1 -Q” which means that only a single mapping was reported for each read, and reads with multiple equally good hits were discarded rather than randomly mapped. The JMP/Genomics 7.1 software (SAS, Cary, NC, USA) was used for data normalization and statistical analysis. The data was normalized using TMM (Trimmed Mean of M component) method [58]. An ANOVA process was conducted as previously described [51]. The liner model was used to test the null hypothesis that expression of a given gene was not different. Specifically, six comparisons (described in the results) were made between the four RILs. We identified genes for which the difference in expression levels within these a priori questions were significantly different (false discovery rate < 0.05) [59] by at least 2-fold.

## Additional files

Additional file 1: The results of mapping reads. (XLSX 14 kb)

Additional file 2: The results of ANOVA analysis. (XLSX 1088 kb)

Additional file 3: SNP positions. (TXT 26 kb)

Additional file 4: Analysis of the boundaries of the D11:24-Mb UHML locus. (PDF 1104 kb) (A) GWAS Manhattan plot of Chr. D11:19–29-Mb, based on the full population and marker set, with locations of select genes discussed in the text labeled. (B) Haplotypes of selected MAGIC RILs with recombinations between the D11:24-Mb and D11:26-Mb loci and the four RILs that were used for RNAseq. Dark grey represents HS26-Paymaster specific (alt) SNPs while white indicates the reference allele. X-axis positions as in (A). (C) Bean plot of the fourteen select recombinant RILs shown in (B) that have only either the D11:24-alt haplotype or the D11:26-alt haplotype. Student’s t-test two-tailed *p*-value for these data is 0.1267. (PDF 1103 kb)

Additional file 5: Primers’ sequences. (XLSX 10 kb)

## Abbreviations

AFIS: Advanced Fiber Information System
ANOVA: Analysis Of Variance
BLUP: Best Liner Unbiased Predictor
DEGs: Differentially Expressed Genes
DPA: Days Post Anthesis
GWAS: Genome Wide Association Study
HVI: High volume instrument
IAA: Indole-3-Acetic Acid
MAGIC: Multi-parent Advanced Generation Inter Cross
MIC: Micronaire
MLM: Mixed Linear Model
nsSNP: non-synonymous Single Nucleotide Polymorphism
QTL: Quantitative Trait Loci
RIL: Recombinant Inbred Line
RT-qPCR: Reverse Transcription quantitative Polymerase Chain Reaction
SCW: Secondary Cell Wall
SEA: gene Set Enrichment Analysis
SFC: Short Fiber Content
SNP: Single Nucleotide Polymorphism
TMM: Trimmed Mean of M component
UHML: Upper Half Mean Length
