# Supplementary figures and images for: Genetic and transcriptomic dissection of the fiber length trait from a cotton (Gossypium hirsutum L.) MAGIC population
Source: BMC Genomics. 2019 Feb 6;20:112. doi: 10.1186/s12864-019-5427-5 (PMC6366115; doi:10.1186/s12864-019-5427-5)

**A**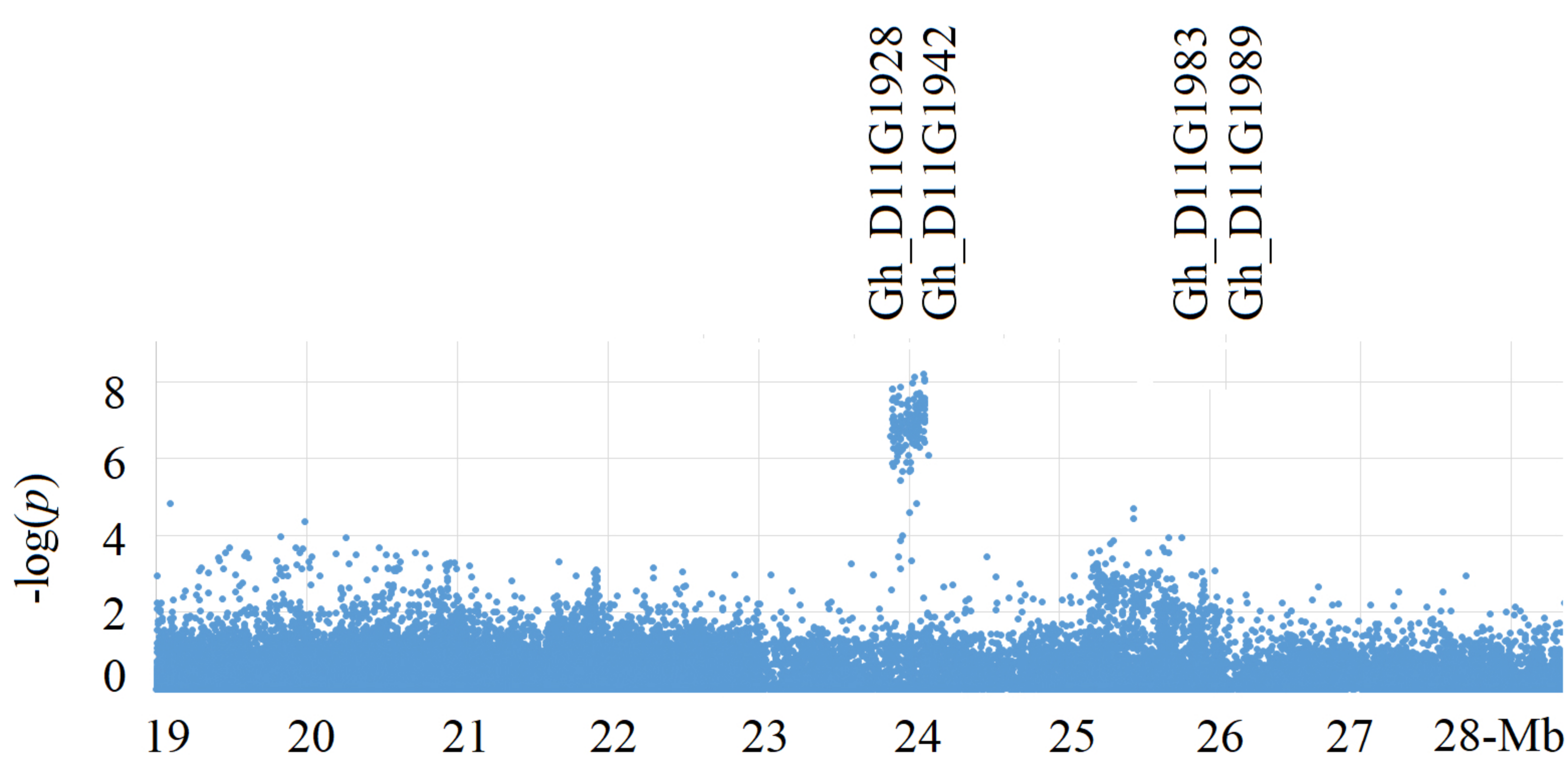**B**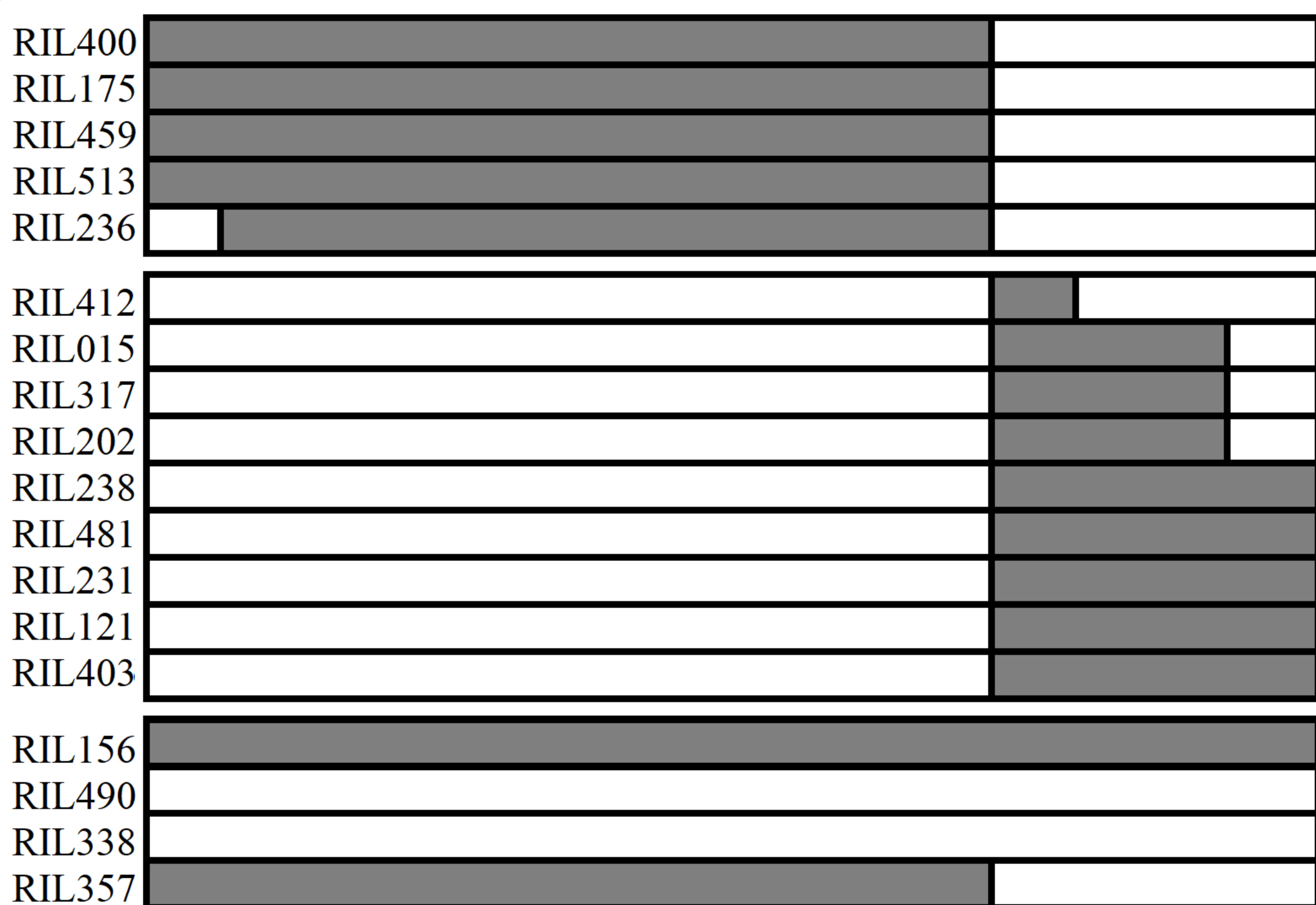**C**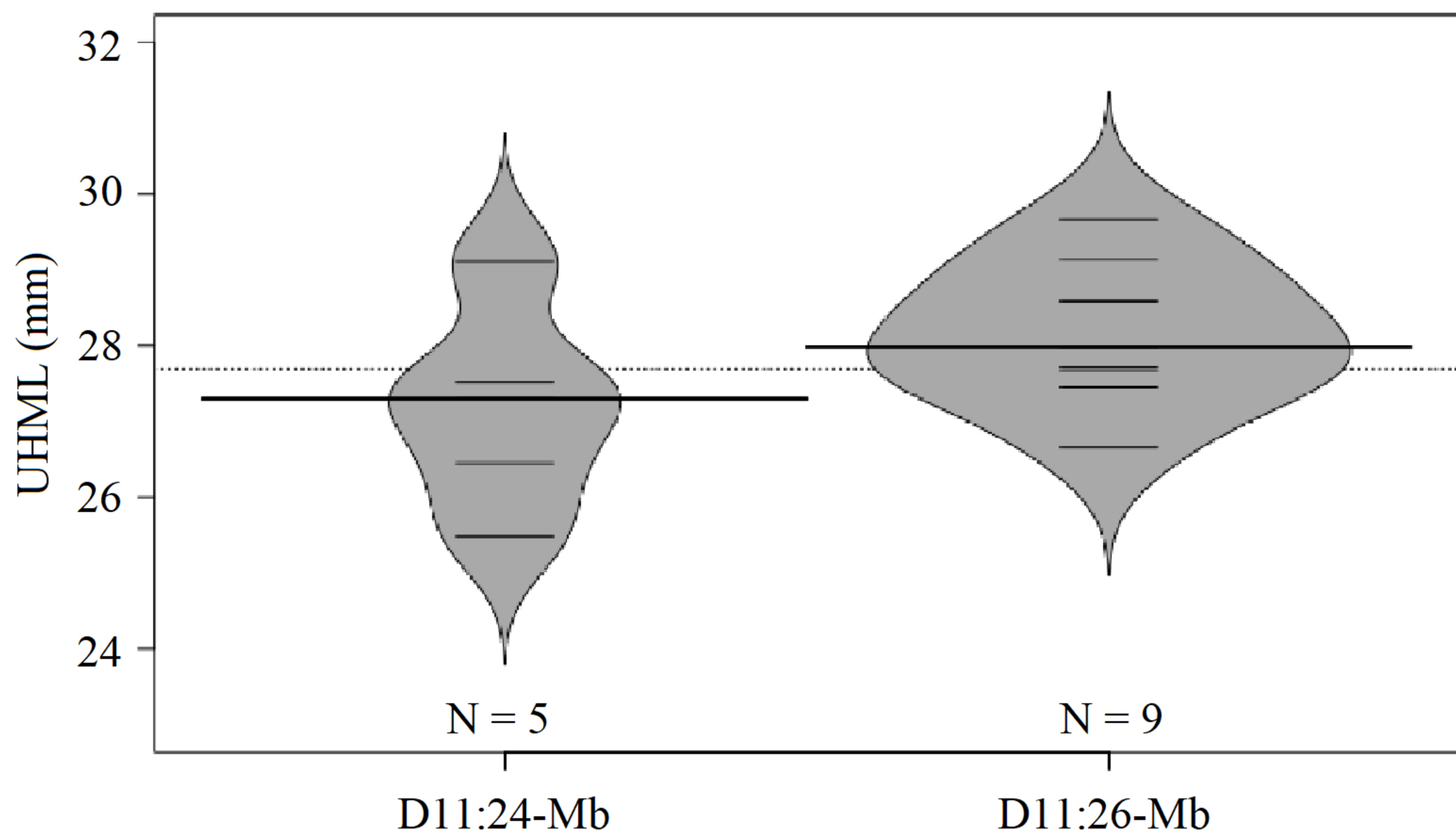

Supplement: Supplementary file 4 — Analysis of the boundaries of the D11:24-Mb UHML locus. (PDF 1104 kb) (A) GWAS Manhattan plot of Chr. D11:19–29-Mb, based on the full population and marker set, with locations of select genes discussed in the text labeled. (B) Haplotypes of selected MAGIC RILs with recombinations between the D11:24-Mb and D11:26-Mb loci and the four RILs that were used for RNAseq. Dark grey represents HS26-Paymaster specific (alt) SNPs while white indicates the reference allele. X-axis positions as in (A). (C) Bean plot of the fourteen select recombinant RILs shown in (B) that have only either the D11:24-alt haplotype or the D11:26-alt haplotype. Student’s t-test two-tailed p-value for these data is 0.1267. (PDF 1103 kb) [file 12864_2019_5427_MOESM4_ESM.pdf]
